# Targeting bone morphogenetic protein receptor 2 sensitizes lung cancer cells to TRAIL by increasing cytosolic Smac/DIABLO and the downregulation of X-linked inhibitor of apoptosis protein

**DOI:** 10.1186/s12964-019-0469-5

**Published:** 2019-11-19

**Authors:** Rachel NeMoyer, Arindam Mondal, Mehul Vora, Elaine Langenfeld, Danea Glover, Michael Scott, Lauren Lairson, Christopher Rongo, David J. Augeri, Youyi Peng, Salma K. Jabbour, John Langenfeld

**Affiliations:** 1 Department of Surgery, Rutgers Robert Wood Johnson Medical School, The State University of New Jersey, New Brunswick, NJ 08903 USA; 2 Department of Genetics, Rutgers University, Piscataway, NJ 08854 USA; 3 RBHS Rutgers Biomedical and Health Sciences, Rutgers University, Piscataway, NJ 08854 USA; 4 Rutgers University, Piscataway, NJ 08854 USA; 5 Ernest Mario School of Pharmacy, Rutgers Translational Science, Rutgers University, Piscataway, NJ 08854 USA; 6 Biomedical Informatics Shared Resources, Rutgers Cancer Institute of New Jersey, New Brunswick, NJ 08903 USA; 7 Department of Radiation Oncology, Rutgers Robert Wood Johnson Medical School, New Brunswick, NJ 08903 USA

## Background

Lung cancer is the leading cause of cancer death in the United States. Despite advances in cancer treatments, 85% of patients diagnosed with lung cancer will succumb to the disease. Bone morphogenetic proteins 2 and 4 (BMP2/4) are highly conserved embryonic proteins required for normal development and regulate the survival, migration, and cell fate decisions of stem cells [1, 2]. BMP signaling is not active in adult lung tissue but is reactivated in lung carcinoma and lung inflammation [2, 3]. The majority of non-small cell lung carcinomas (NSCLC) highly overexpress the BMP2 ligand [4]. BMP signaling in lung cancer regulates cell survival, migration, proliferation, stemness, angiogenesis, and ligand overexpression and is correlated with a worse prognosis [3, 5–8]. BMP signaling stimulates tumorigenesis in many carcinomas including prostate [9], breast [10, 11], pancreatic [12], melanoma, and sarcoma [13]. The BMP receptors are expressed in all NSCLC and inactivating mutations are infrequent [14].

There are over 20 BMP ligands that signal through serine/threonine kinases. The BMP ligands bind to the BMP type I receptors (ALK2, ALK3, or ALK6) [15], which are phosphorylated by the constitutively active BMP type 2 receptors (BMPR2, ActR-IIA, ActR-IIB) [15]. The BMP receptor complex then phosphorylates Smad 1/5 [16], which then translocates to the nucleus, transcriptionally regulating downstream targets including the inhibitor of differentiation proteins (ID1, ID2, and ID3) [17, 18].

The BMP signaling cascade also regulates Smad 1/5-independent mechanisms. Smad 1/5-independent signaling occurs by the binding of proteins to the cytosolic tail of the BMP receptor. BMP regulation of cancer cell survival involves the regulation of X chromosome-linked inhibitor of apoptosis protein (XIAP) and transforming growth factor beta (TGFβ) activated kinase 1 (TAK1), an evolutionary conserved Smad 1/5-independent signaling pathway [19–21]. During embryonic development, BMPR2 regulates XIAP, which leads to the activation of TAK1 [22]. Both XIAP and TAK1 are potent inhibitors of cell death in cancer cells. XIAP inhibits apoptosis by binding to and inactivating effector caspases 3, 7, and 9 [23]. XIAP also functions as an E3 ligase inducing the degradation of caspases via the proteasome system [24]. TAK1 inhibits cell death by activating nuclear factor-kappa beta (NF-κB) [25] and inhibits reactive oxygen species (ROS) production [26]. XIAP is being targeted as a cancer therapeutic because its inhibition of caspases promotes resistance to cancer therapeutics that induce apoptosis including tumour-necrosis factor (TNF)-related apoptosis-inducing lingand (TRAIL) and various chemotherapeutics [23, 27, 28].

Several generations of small molecule inhibitors of BMP receptors have been derived from the same pyrazolo [1,5-*a*] pyrimidine core [29–31]. JL5 is an analog of DMH2, with improved pharmacokinetic properties compared to DMH2, that has been demonstrated to cause tumor regression of lung cancer xenografts [14, 20]. JL5 and DMH2 both cause a decrease in the expression of XIAP and a decrease in TAK1 activity [14, 20]. The mechanism by which the inhibition of BMP signaling decreases XIAP expression has not been fully elucidated. DMH2 and JL5 cause greater inhibition of BMP signaling, induce more cell death, and decrease expression of XIAP compared to the BMP inhibitors DMH1 and LDN [14, 20]. These BMP inhibitors all have potent inhibition of BMP type I receptors. JL5 and DMH2 demonstrate inhibition of BMPR2 while DMH1 and LDN have no activity for BMPR2. It is unknown whether the enhanced activity of JL5 is caused by its inhibition of BMPR2 smad-independent signaling.

In this study, we show that the BMP inhibitor JL5 enhances cell death of TRAIL and the Smac mimetic AEG 40730 treated lung cancer cells. JL5 enhances apoptosis by inducing the downregulation of XIAP through its inhibition of BMPR2 receptor function. Knockdown of BMPR2, but not BMP type I receptors, increase cytosolic Smac/DIABLO, which is a known inhibitor of XIAP. These studies show that BMPR2 regulates cell survival signaling pathways not mediated by type I receptors and that targeted inhibition of BMPR2 may enhance apoptotic cell death of cancer therapeutics.

## Methods

### Cell culture and reagents

The H1299 and A549 lung cancer cell lines (ATCC) were maintained in Dulbecco’s Modified Eagle’s medium (DMEM, Sigma Aldrich, St Louis, MO, USA) supplemented with 5% fetal bovine serum [3]. JL5 and DMH2 were synthesized by the David Augeri laboratory, Rutgers School of Pharmacy. TRAIL was purchased from Thermo Fisher Scientific, Trolox from Cayman Chemical, Z-VAD-FMK from Selleckchem, and AEG 40730 from Tocris. DMH1 was purchased from Selleckchem (Houston, TX). Constitutively active ALK3 and ALK6 constructs were a gift from Joan Massague (Memorial Sloan Kettering Cancer Center, New York, New York) and the pcDNA3-XIAP-Myc K322/D28 was purchased from Addgene (Watertown, Massachusetts).

### Immunoblot analysis

Western blot analysis was performed as previously reported [3]. In brief, total cellular protein was generated using RIPA buffer and concentration was measured using the BCA assay. Protein was separated by SDS-PAGE and transferred to nitrocellulose. The blots were incubated overnight at 4 °C with the primary antibody. The primary antibodies that were used were rabbit monoclonal anti-Smac/DIABLO, rabbit monoclonal anti-cytochrome c, rabbit monoclonal anti-cIAP1, rabbit monoclonal anti-pTAK1, rabbit monoclonal XIAP, rabbit monoclonal anti-activated caspase-3, rabbit monoclonal anti-activated caspase-8, rabbit monoclonal anti-PARP (Cell signaling Technology, Danvers MA), rabbit monoclonal anti-ID1(Calbioreagents, San Mateo, CA), rabbit anti-actin, an affinity isolated antigen specific antibody (Sigma, Saint Louis, MO), and rabbit polyclonal anti-GAPDH (Sigma, St. Louis, MO).

### Cell viability

Lung cancer cells were plated into 6-well plates and treated the next day for the designated period of time. Cells were trypsinized and the number of live and dead cells were determined using the Vi-CELL cell analyzer (Beckman Coulter), which analyzed 500 cells per sample and utilized trypan blue dye exclusion to determine dead cells.

### Transient knockdown and transfection

Validated select siRNA was used to knockdown the expression of XIAP, BMPR2, ALK3, and ALK6 (Life Technologies). The ID numbers for the siRNA are: XIAP (S1456), ALK3 (s281), ALK6 (s2042), and BMPR2 (s2044 and s2045). Silencer Select negative control siRNA (4390843) was used to evaluate selectivity. Silencer Select negative controls do not target any gene product and have no effect on cell proliferation or viability. Transfections of the siRNA were performed using Lipofectamine® RNAiMAX Reagent (Invitrogen, Carlsbad, CA, USA) according to manufacturer’s protocol. Briefly, H1299 and A549 lung cancer cell lines were seeded for 24 h (h) up to 70–80% confluence at the time of transfection. 150 μl of Opti-MEM® Medium was used to dilute 9 μl of Lipofectamine® RNAiMAX Reagent, control siRNA and target siRNA. Diluted Lipofectamine® RNAiMAX Reagent was mixed with diluted siRNA in 1:1 ratio and incubated for 5 min (min) at room temperature to obtain the RNA-lipid complex. The cells were incubated in siRNA-lipid complexes for 24 h at 37 °C. After 24 h, the media was changed to fresh media and the transfected cells were used for further experiments. Cells were transfected with 30 nM ALK3, 20 nM ALK6, 30 nM XIAP, and 6 nM BMPR2.

### Cytosol extraction

Cytosolic protein extraction was performed using Mitochondria/Cytosol fractionation kit (Enzo Life Sciences, NY, USA). Briefly, 750,000 cells/well were seeded in 6-well plates for 24 h. The cells were treated with TRAIL and JL5 at designated times. After treatment, the cells were trypsinized, pelleted and washed twice with cold phosphate-buffered saline (PBS) and centrifuged at 600 x g for 5 min at 4 °C. Supernatant was removed and cell pellets were resuspended in 100 μl of ice-cold Cytosol Extraction Buffer Mix containing DL-Dithiothreitol (DTT) and Protease Inhibitors. After a 10 min incubation on ice, cells were homogenized. The homogenates were collected to a fresh 1.5 ml tube and centrifuged at 700 x g for 10 min at 4 °C. The supernatant was collected in a 1.5 ml tube and centrifuged at 10,000 x g for 30 min. at 4 °C. The supernant was collected as the cytosolic fraction and used for further experiments.

### Reactive oxygen species (ROS) measurements

Intracellular reative oxygen species (ROS) development after treatment of JL5 and TRAIL alone or in combination were measured by the total ROS detection kit (Enzo Life Sciences) according to the manufacturer’s protocol. Briefly, H1299 cells were seeded in a 6-well plate at a density of 750,000 cells/well. After 24 h, cells were treated with DMSO, 2.5uM JL5 and 50 ng/ml TRAIL alone or in combination for 3 h, 24 h and 48 h. At the end of the treatment, cells were trypsinized and then stained with ROS detection solution. Stained cells were incubated in the dark at 37 °C for 30 min. The results were monitored by using a flow cytometer (BD Biosciences).

### TUNEL assay

Deoxyribonucleic acid (DNA) double-strand breaks (DSB) after treatment were analyzed using FlowTACS In Situ TUNEL-based apoptosis detection kit (Trevigen) according to the manufacturer’s protocol. Briefly, 750,000 cells/well were seeded in 6-well plates for 24 h. The cells were treated with JL5, DMH2, TRAIL and JL5 and TRAIL combination at designated times. After treatment, cells were trypsinized and the cell pellet was fixed with 4% formaldehyde and permeabilized with cytonin for 30 min. After washing with labeling buffer, cells were resuspended in the labeling reaction mix and incubated for 1 h at 37 °C. Then the cells were stained with strep-fluorescein solution for 10 min at 37 °C. The samples were analyzed by using flow cytometry (LSRII, BD Biosciences).

### Immunofluorescence staining

H1299 cells at 450,000 cells/well concentration were seeded for 24 h onto microscope cover glasses in a 6-well plate. Next, cells were treated with 2.0 μM DMH1 or 2.5 μM JL5 for 24 h. After treatment, cells were fixed with 4% formaldehyde and permeabilized with 0.5% triton-X. After blocking with CAS-block for 1 h, cells were stained with anti-BMPR2 antibody (Sigma-Aldrich) for 1 h at room temperature. Cells were washed with PBS and stained with Alexa Flour 488 conjugated secondary antibody for 1 h at room temperature. After washing with PBS, the nuclei were counterstained with 4′,6-diamidino-2-phenylindole (DAPI) (Sigma-Aldrich) for 10 min. Coverslips were then washed with PBS, rinsed with Mili-Q water and mounted with a mounting media. After drying, cells were observed under a fluorescence microscope (Nikon eclipse TE300).

### Quantification of gene expression

Quantitative polymerase chain reaction (PCR) was performed for BMPR1A (ALK3), BMPR1B (ALK6), and BMPR2 following knockdown with small interfering ribonucleic acid (siRNA) as previously reported [20, 32]. In brief, RNA was extracted using the RNeasy kit (Quagen, Valencia, CA) and then treated with DNAse. cDNA was generated using Advantage RT for PCR kit (BD Bioscience, Clontech, Palo Alto). Quantitative PCR was performed with the Stratage Mx3005p (Agilent Technologies) and validated Taq-Man primers according to the manufacturer’s specifications (Applied Biosystems, Foster City, CA). Reference numbers used were: GAPDH (Hs99999905_m1), BMPR1A (ALK3) (Hs00831730_s1), BMPR1B (ALK6) (Hs00176144_m1), BMPR2 (Hs00176148_m1). Negative control included all reagents except cDNA. Expression was normalized to GAPDH using the formula 2 ^ΔCT^.

### BMPR2 (*daf-4*) response to JL5 in *Caenorhabditis elegans* (*C. elegans*)

#### Assessing BMP activity using *spp-9::GFP* reporter

Animals were age synchronized and treated with drug at the L1 stage at the indicated concentrations for JL5. Animals were then grown at 20 °C until the L4 stage. Live animals at the L4 stage were mounted on 2.5% (w/v) agarose and anesthetized using 10 mM levamisole. Animals were imaged at 5x magnification on a standard epifluorescent microscope. The average total intensity was calculated. Imaging quantification was performed using the open-source Fiji Software for each individual animal using the “Segmented Line” tool. A minimum of 60 animals were quantified for each condition performed twice. A one-way analysis of variation (ANOVA) was performed to compare differences in mean intensity across conditions.

#### Localization experiments for *daf-4::GFP*

Animals were age synchronized and treated with drug at the L1 stage at the indicated concentrations of JL5. Animals were then grown at 20 °C until the L4 stage. Live animals at the L4 stage were mounted on 25 (w/v) agarose and anesthetized using 10 mM Levamisole. Animals were imaged at 63x magnification on a laser spinning disc confocal microscope (Zeiss). Either the 3rd or 4th cell (from the anterior end) of the intestine was imaged. A minimum of 60 animals were quantified for each condition performed twice. An ANOVA was performed to compare differences in mean intensity across conditions.

### Statistical analysis

The mean of the control group as compared to the mean of each treated group using a paired student t-test assuming unequal variances. Differences with *p* values < 0.05 were considered statistically significant.

## Results

### JL5 enhances cell death of TRAIL treated lung cancer cells

**Fig. 1:**
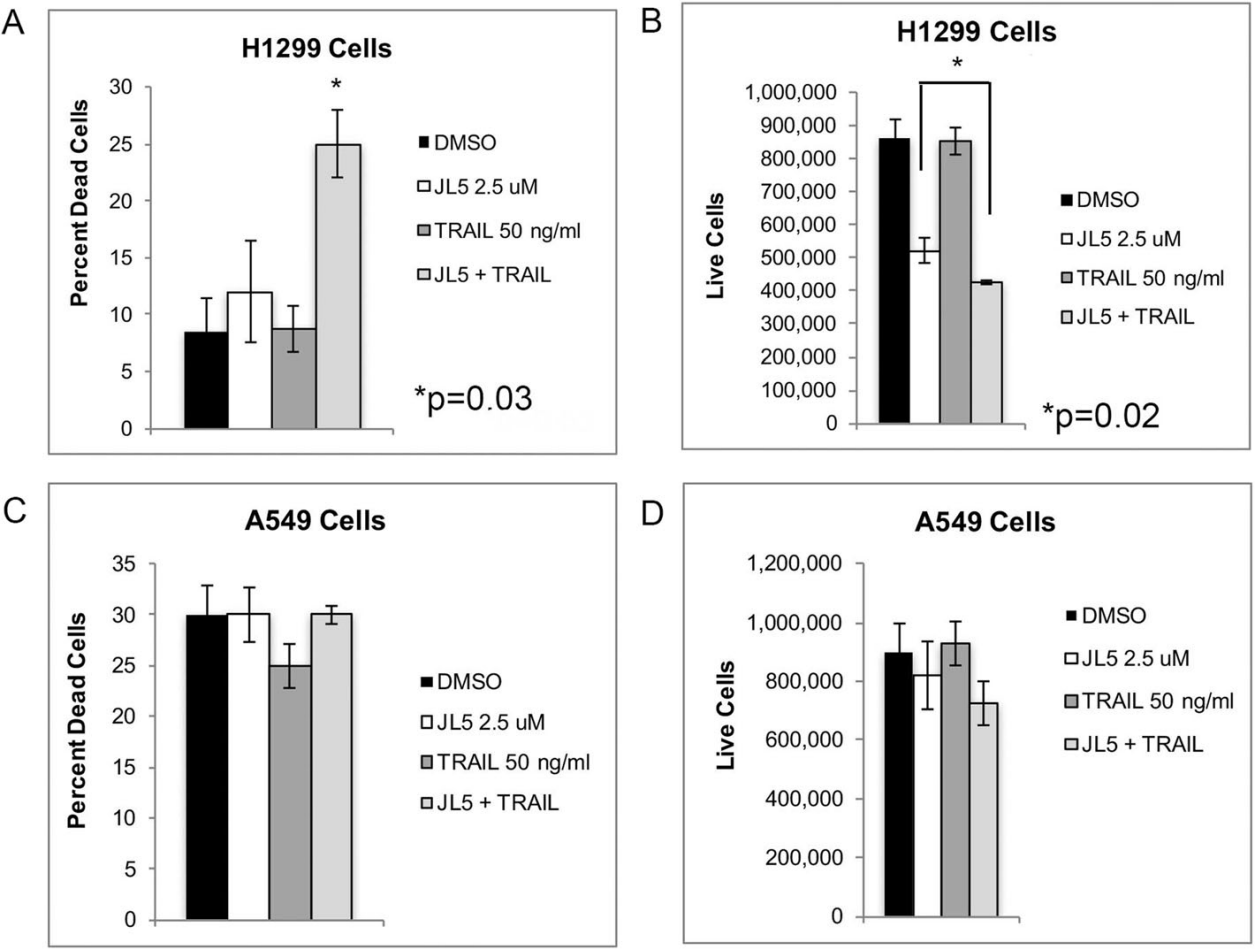
JL5 enhances cell death induced by TRAIL. H1299 cells (**a**-**b**) and A549 cells (**c**-**d**) were treated with JL5 and TRAIL alone and in combination for 24 h and the percent dead and number of live cells determined. Significantly more cell death occurred in H1299 cells treated with JL5 and TRAIL than either agent alone (**c**-**d**). In A549 cells, JL5 and TRAIL alone or in combination had little effect on cell death after 24 h. Data represents the mean percentage of dead cells and the number of live cells of 4 independent experiments

Since JL5 decreases the expression of XIAP [20], a known inhibitor of apoptosis, we examined whether JL5 enhanced cell death induced byTRAIL. TRAIL induces extrinsic apoptosis by activating caspase-8, which cleaves and activates the executioner caspase-3 [33]. H1299 cells have a p53 mutation and are sensitive to BMP inhibitors [20]. A549, a TRAIL resistant cell line [34], has a K-ras mutation and is less sensitive to BMP inhibitors compared to H1299 cells [20]. TRAIL alone demonstrated no effect on cell death in either the H1299 or A549 cells (Fig. 1a-d). The combination of JL5 and TRAIL used simultaneously caused significantly more cell death than either agent alone, in H1299 cells (Fig. 1a-b) but not in A549 cells (Fig. 1c-d).

### JL5 enhances TRAIL-induced caspase-3 activation in H1299 cells

**Fig. 2:**
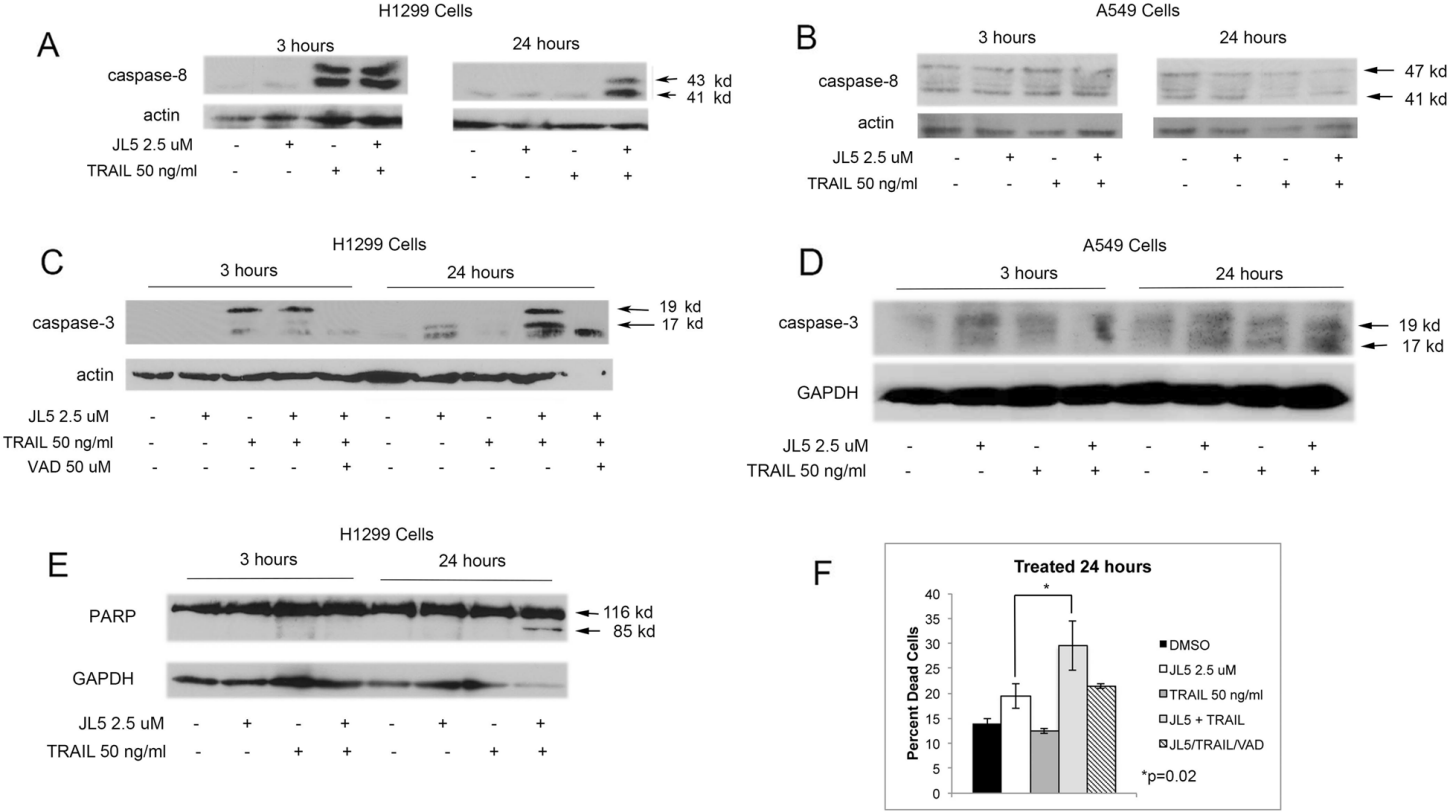
JL5 enhances TRAIL activation of caspase-3. **a**-**b** Immunoblot analysis of activated caspase-8 in (**a**) H1299 and (**b**) A549 cells treated with JL5 and TRAIL alone and in combination for 3 and 24 h. Trail induced cleavage of procaspase-8 into 41 kDa and 43 kDa activated fragments in H1299 cells but not in A549 cells. (C) Western blot analysis in H1299 demonstrated that caspase-3 is fully processed to its active 17 kDa fragment at 24 h only in cells treated with both JL5 and TRAIL. **c** The caspase inhibitor Z-VAD-FMK (VAD) prevented JL5/TRAIL processing of caspase-3 into its 17 and 19 kDa fragments. **d** TRAIL and/or JL5 did not activate caspase-3 in A549 cells. **e** Western blot analysis demonstrated PARP cleavage into the 85 kDa fragment only in H1299 cells treated with both JL5 and TRAIL for 24 h. **f** VAD inhibited cell death induced by JL5 in combination with TRAIL in H1299 cells. Data represents the mean percentage of dead cells of 4 independent experiments

To elucidate the mechanisms by which JL5 enhances TRAIL induced cell death in H1299 cells, we examined the activation of the caspases. TRAIL binds the TRAIL receptor forming a death-inducing signaling complex leading to self-cleaving of procaspase 8 that activates effector caspase-3. TRAILinduced cleavage of caspase-8 within 3 h after treatment in H1299 cells, indicates that the TRAIL receptor complex was activated in these cells (Fig. 2a). Consistent with a report, TRAIL does not activate caspase-8 in A549 cells (Fig. 2b) [34]. In H1299 cells, caspase-3 was cleaved into the 19 kDa fragment but was not fully processed into the 17 kDa form, indicating that caspase-3 was not fully active [35, 36] (Fig. 2c). At 24 h, caspase-3 was cleaved into both the 17 kDa and 19 kDa fragments, but only in H1299 cells that were treated with both JL5 and TRAIL (Fig. 2c). The pan-caspase-3 inhibitor Z-VAD-FMK (VAD), prevented the processing of caspase-3 into the 17 kDa and 19 kDa fragments (Fig. 2c). In A549 cells, treatment with JL5 and TRAIL, either alone or in combination, did not induce the activation of caspase-3 (Fig. 2d). Long immunoblot exposure showed low levels of activated caspase-3 that did not increase with treatment (Fig. 2d). Processing caspase-3 to its 17 kDa fragment is required for full apoptotic activity [36]. Activation of caspase-3 cleaves its downstream target PARP into an 85 kDa fragment [36]. Only H1299 cells treated with both JL5 and TRAIL after 24 h demonstrated cleavage of PARP (Fig. 2e). VAD partially inhibited JL5 and TRAIL-induced cell death (Fig. 2f) indicating that the activation of the caspases is involved in the cell death process. These data suggest that JL5 enhances cell death of TRAIL by increasing the full processing of caspase-3 to its activated form.

### JL5 induced decrease in XIAP expression enhances TRAIL induced caspase-3 activation

**Fig. 3:**
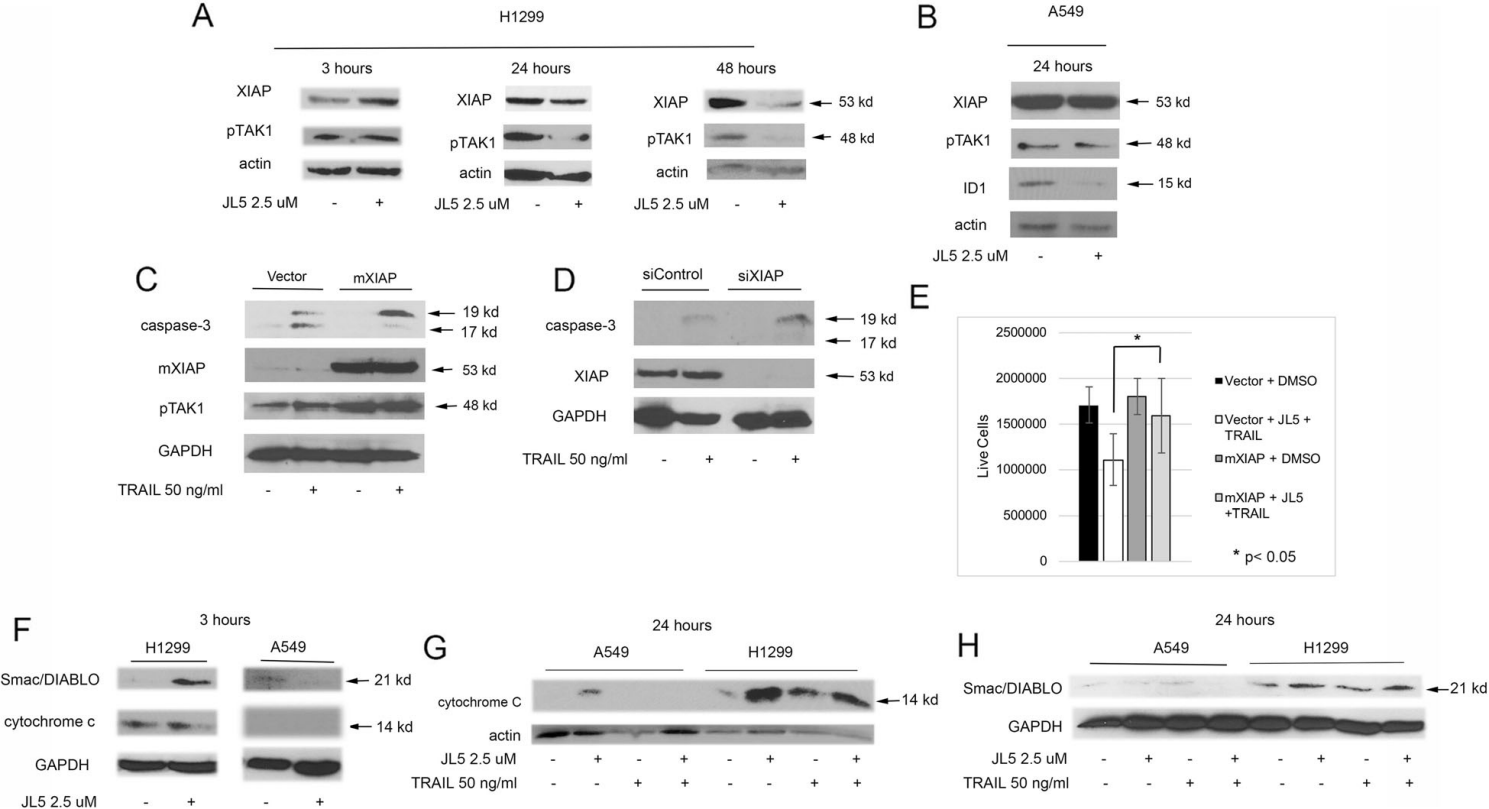
JL5 downregulation of XIAP enhances TRAIL induced caspase-3 dependent cell death in H1299 cells and JL5 increases cytosolic Smac/Diablo. **a**-**b** Immunoblot demonstrated that JL5 decreases expression of XIAP in H1299 cells at 24–48 h but has no effect on (**b**) A549 cells at 24 h. **a,c** TAK1 activity directly correlates with the expression of XIAP. **c** H1299 cells were transfected with mXIAP or control vector, then treated with TRAIL for 3 h. Immunoblot demonstrates mXIAP inhibited TRAIL processing of caspase-3 to its 17 kDa form. **d** H1299 cells were transfected with siRNA targeting XIAP for 48 h then treated with TRAIL for 3 h. Immunoblot demonstrates that knockdown of XIAP enhanced TRAIL induced processing of caspase-3 to its 19 kDa fragment. **e** H1299 cells were transfected with mXIAP or control vector for 24 h then treated with JL5 together with TRAIL for 24 h and cell counts performed. Data represents the mean of 4 independent experiments showing mXIAP inhibited cell death induced by JL5 in combination with TRAIL. **f** Western blot of cytosol demonstrating that JL5 increases cytosolic Smac/DIABLO as early as 3 h in the H1299 cells but not the A549 cells. **g**-**h** Immunoblot of cytosol of cells treated with JL5 and TRAIL alone and in combination for 24 h. **g** Both JL5 and TRAIL increase cytosolic cytochrome c, while (**h**) JL5 alone increased cytosolic Smac/Diablo in the H1299 cells but not in the A549 cells

We previously reported that DMH2 and JL5 decrease the expression of XIAP in H1299 cells but the BMPR1 inhibitors DMH1 and LDN-193189 (LDN) do not [14, 20]. To further examine the regulation of XIAP by BMP inhibitors, we examined the timing in which JL5 induced the decrease in expression of XIAP. JL5 did not decrease the expression of XIAP after 3 h but a decrease in expression of XIAP was identified at 24 and 48 h in H1299 cells (Fig. 3a). We found that the downregulation of pTAK1 correlated to the decrease in expression of XIAP, confirming previous reports that TAK1 is downstream of XIAP (Fig. 3a) [20, 22]. In A549 cells, JL5 did not decrease the expression of pTAK1 or XIAP but did decrease ID1 after 24 h (Fig. 3b). This indicated that in the A549 cells, Smad 1/5-dependent regulation of ID1 was inhibited by JL5 but not the Smad 1/5-independent downregulation of XIAP. Furthermore, the data suggest that the downregulation of XIAP may be needed for JL5 to enhance cell death with TRAIL.

XIAP inhibits caspase-3 apoptotic activity by binding the activated 19 and 17 kDa fragments as well as inhibiting its processing to the 19 and 17 kDa forms [35, 36]. Downregulating the expression of XIAP can overcome resistance to TRAIL [35, 36]. To determine whether the decreased expression of XIAP is the mechanism by which JL5 enhances apoptosis, H1299 cells were transiently transfected with XIAP that had its ubiquitination sites removed (mXIAP) [37], or vector control, and treated with TRAIL for 3 h. Processing of caspase-3 to its active 17 kDa form was less efficient in cells transfected with mXIAP as compared to cells transfected with vector control (Fig. 3c). Cells transfected with mXIAP also had an increased expression of pTAK1, confirming an increased activity of XIAP (Fig. 3c). The knockdown of XIAP with siRNA enhanced TRAIL induced activation of caspase-3 after 24 h compared to controls (Fig. 3d). Forced expression of mXIAP inhibited cell death induced by JL5 alone and in combination with TRAIL (Fig. 3e). These studies demonstrate that JL5 enhances apoptotic cell death induced by TRAIL and involves a decrease in the expression of XIAP.

### JL5 causes the release of Smac/DIABLO into the cytosol

Increased permeability of the mitochondrial outer membrane allows the release of the proapoptotic agents Smac/DIABLO and/or cytochrome c into the cytosol. Cytosolic Smac/DIABLO binds XIAP with high affinity inhibiting its anti-apoptotic effects on activated executionary caspases-3 and 7 leading to apoptosis [36]. Smac/DIABLO can also induce the ubiquitination and proteasomal degradation of XIAP [38]. Since JL5 decreases expression of XIAP and enhances apoptosis, we examined whether it increases cytosolic Smac/DIABLO and/or cytochrome c. In H1299 cells, JL5 increased cytosolic Smac/DIABLO within 3 h, which persisted for up to 24 h (Fig. 3f, h). Both JL5 and TRAIL increased cytosolic cytochrome c in H1299 cells after 24 h (Fig. 3g). TRAIL did not increase cytosolic Smac/DIABLO expression after 24 h (Fig. 3h). JL5 did not increase cytosolic Smac/DIABLO or cytochrome c induced by TRAIL (Figures G-H). In A549 cells, JL5 and TRAIL had little effect on cytosolic cytochrome c or Smac/DIABLO expression in the time points examined (Fig. 3g-h). Since JL5 increases cytosolic Smac/DIABLO expression prior to the decrease in the expression of XIAP suggests that increasing cytosolic Smac/DIABLO expression may be a mechanism by which JL5 downregulates XIAP in H1299 cells.

### Knockdown of BMPR2 but not BMP type 1 receptors increases cytosolic Smac/DIABLO levels

**Fig. 4:**
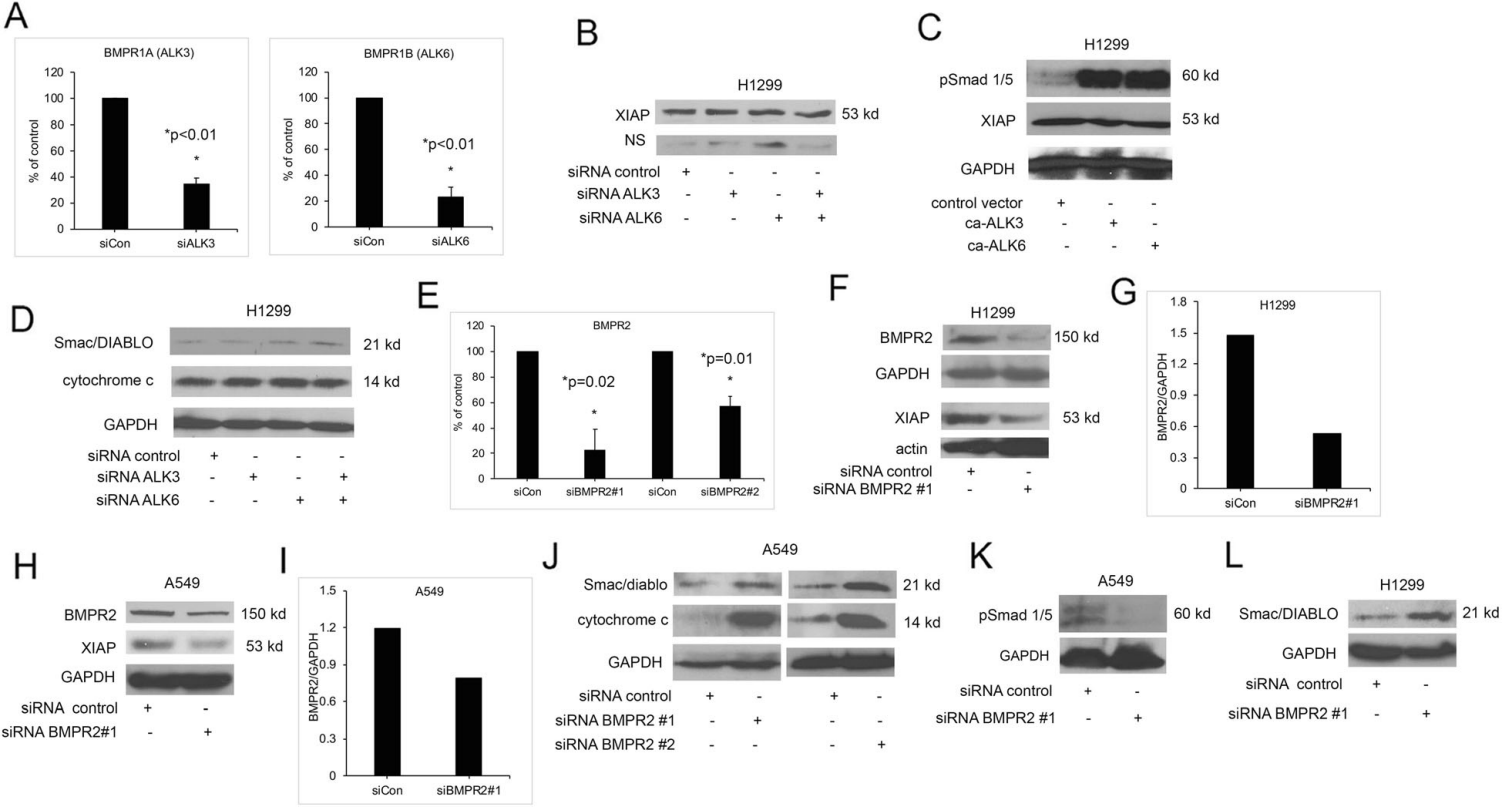
Knockdown of BMPR2 but not BMP type 1 receptors decreases expression of XIAP and increases cytosolic Smac/DIABLO and cytochrome c. **a** Quantitative PCR demonstrating a decrease in expression of ALK3 and ALK6 following siRNA knockdown in H1299. Data represents the mean of 2 independent experiments done in duplicate reported as the percent of siRNA control. **b** Western blot analysis of cells transfected with siRNA targeting alk3 or alk6 alone or in combination for 24 h showing no change in XIAP expression. NS is a nonspecific band used as a loading control. **c** Western blot analysis of H1299 cells that were transfected with constitutively active ALK3 or ALK6 showing activation of Smad-1/5 but no change in XIAP expression. **d** Western blot analysis showing that knockdown of ALK3 or ALK6 alone or in combination does not increase cytosolic expression of Smac/DIABLO or cytochrome c. **e** Quantitative PCR using two different siRNA for controls and BMPR2 demonstrating decreased BMPR2 RNA expression in H1299 cells. Data represents the mean of 2 independent experiments done in duplicate reported as the percent of the siRNA control. **f** Western blot analysis of H1299 cells transfected with control or BMPR2 siRNA showing a decrease in XIAP expression. **g** The actin and BMPR2 bands were quantified using Image J and reported as the ratio of BMPR2/actin. **h** Western blot analysis of A549 cells transfected with control or BMPR2 siRNA showing a decrease in XIAP expression. **i** The GAPH and BMPR2 bands were quantified using Image J and reported as the ratio of BMPR2/GAPDH. **j** Western blot analysis of cytosol of A549 cells following knockdown of BMPR2 using two different siRNA demonstrating an increase in Smac/DIABLO and cytochrome c expression. **k** Western blot analysis showing knockdown of BMPR2 decreases BMP signaling. **l** Western blot of H1299 cells following BMPR2 knockdown demonstrating an increase in cytosolic Smac/DIABLO. **b**-**l** Studies were done at least 3 times with similar results

We hypothesized that JL5 induced the downregulation of XIAP and increased cytosolic Smac/DIABLO by its inhibition of BMPR2 and not the BMP type 1 receptors. To test this hypothsis, we knocked down the BMP type 1 receptors and BMPR2 using siRNA and examined the expression of XIAP and changes in cytosolic Smac/DIABLO and/or cytochrome c. BMPRIA (ALK3) and BMPRIB (ALK6) are the primary type 1 receptors receptors regulating BMP signaling. We previously published that knockdown of ALK3 and ALK6 expression in H1299 cells decreased BMP signaling [32]. We confirmed that the siRNA decreased RNA expression of ALK3 and ALK6 in H1299 cells (Fig. 4a). The low protein expression of ALK3 and ALK6 made it difficult to effectively determine changes in protein levels by Western blot analysis. The knockdown of ALK3 or ALK6 alone and in combination did not change the expression of XIAP (Fig. 4b). Transfection of a consitutively active ALK3 (ca-ALK3) or ALK6 (ca-ALK6) into H1299 cells showed the expected increase in the phosphorylation of Smad-1/5 but had no effect on the expression of XIAP (Fig. 4c). Knockdown of alk3 or alk6 alone or in combination had no effect on the amount of Smac/DIABLO or cytochrome c in the cytosol (Fig. 4d). These studies suggest that JL5 induced downregulation of XIAP expression and increased cytosolic Smac/DIABLO is not mediated by inhibiting the BMP type I receptors.

We next examined whether JL5 regulation of XIAP and the increase in cytosolic Smac/DIABLO and/or cytochrome c was mediated by the inhibition of BMPR2. Quantitative PCR showed two different siRNA decreased BMPR2 RNA expression (Fig. 4e). The siRNA caused a 70 and 50% reduction in BMPR2 protein expression in comparison to control in H1299 and A549 cells respectively (Fig. 4 f-i). Consistent with our prior report, the knockdown of BMPR2 decreased XIAP expression in both H1299 (Fig. 4f) and A549 cells (Fig. 4h). Both BMPR2 siRNAs increased cytosolic Smac and cytochrome c in A549 cells (Fig. 4j). Knockdown of BMPR2 was associated with a decrease in Smad 1/5 activity, confirming downregulation of BMP signaling (Fig. 4k). Knockdown of BMPR2 in H1299 cells also caused an increase in the expression of Smac/DIABLO in the cytosol (Fig. 4l), however, no clear increase in cytoplasmic cytochrome c was detected at this time point (Fig. 4l). These studies suggest that JL5 mediates the downregulation of XIAP and increases cytosolic Smac/DIABLO by inhibiting BMPR2.

### JL5 causes cytoplasmic trapping of BMPR2 in lung cancer cells and in *C. elegans*

**Fig. 5:**
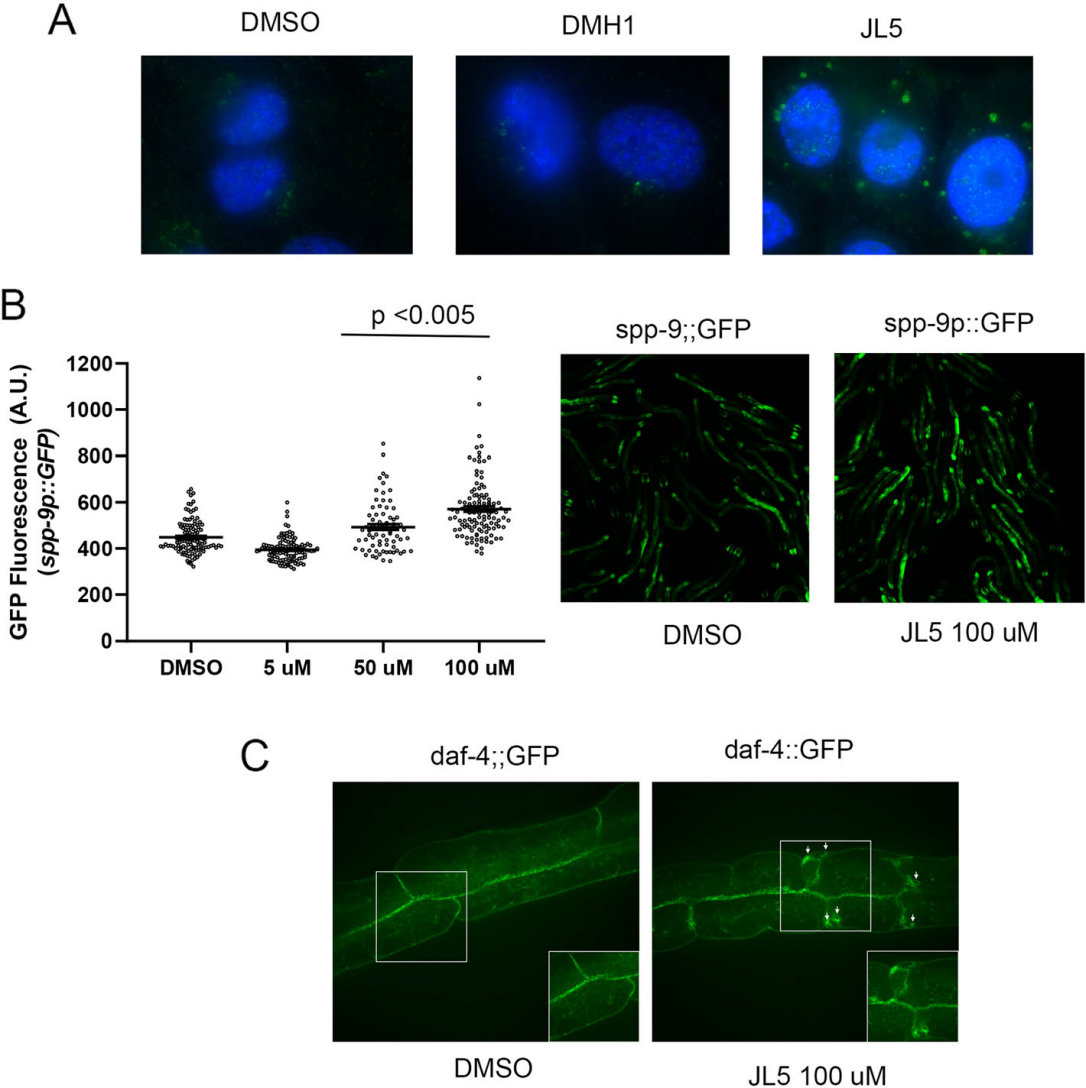
JL5 induces cytosolic trapping of BMPR2 in lung cancer cells and *C. elegans*. **a** H1299 cells were treated with DMSO, DMH1 2.0 μM, or JL5 2.5 μM for 24 h and immunofluorescent imaging performed for BMPR2 (green) and nuclear staining with DAPI. Only cells treated with JL5 demonstrated BMPR2 trapped within cytoplasmic vesicles. Studies were performed at least 3 times with the same results. **b** Various concentrations JL5 were applied to L1 *C. elegans* bearing a germline-integrated *spp-9::GFP* transgene. After 72 h animals were imaged at 5x magnification on a standard epifluorescent microscope. Imaging quantification was performed using the open-source Fiji Software. Graphs represent population spread with mean +/− SEM. A minimum of 60 animals were quantified for each condition. A one-way ANOVA was performed to compare differences in mean intensity across conditions. JL5 increased GFP fluorescent intensity indicating a decrease in BMP signaling. **c** JL5 traps BMPR2 (*daf-4*) within an intracellular compartment. *C. elegans* bearing a *daf-4::GFP* (BMPR2) transgene were treated with DMSO or JL5 for 72 h and live animals examined by confocal microscopy. Arrows demonstrate abnormal *daf-4::GFP* (BMPR2) accumulation within vesicles close to the basolateral membrane. Sixty live animals were examined for each condition, which were performed twice

One difficulty explaining that JL5 mediates its anti-tumorigenic effects through its inhibition of BMPR2 is that JL5 has an IC50 for BMPR2 kinase activity of 8 μM and we are treating the cells with 2.5 μM. We hypothesized that JL5 may be affecting BMPR2 function by mechanisms other than its inhibition of its kinase activity. Trafficking of BMPR2 from the plasma membrane into the cytosol then back to plasma membrane is required to maintain normal function. Mislocalization of BMPR2 from the plasma membrane to the cytoplasm leads to the inhibition of BMPR2 signaling [39, 40]. We examined whether JL5 altered the localization of BMPR2. We utilized the BMP type I receptor inhibitor DMH1 as a control since it does not inhibit BMPR2 kinase activity or decrease the expression of XIAP. By immunofluorescent imaging, we found that JL5, but not DMH1, causes intracellular accumulation of BMPR2 in the H1299 cells (Fig. 5a).

We examined whether the effects of JL5 on BMPR2 localization are conserved in *C. elegans*. The intracellular trafficking and recycling of the BMPR2 has been studied in great detail in *C. elegans* [41]. The nematode BMPR2, *daf-*4, is internalized utilizing a clathrin-independent mechanism and recycles back to the plasma membrane via the recycling endosome [41]. We first showed that BMP signaling in the worm can be inhibited with JL5 (Fig. 5b). Microarray studies conducted in *C. elegans* have identified BMP regulated genes [42]. The *spp-9* gene is negatively regulated by BMP signaling; mutants in the BMP signaling pathway exhibit increased *spp-9::GFP* expression in live animals bearing this transgene [42]. In order to determine whether JL5 inhibits BMP signaling in *C. elegans*, animals bearing a germline-integrated *spp-9::GFP* transgene were treated with varying concentrations of the drugs from early L1 stage and *spp-9::GFP* expression was assayed at L4 stage worms (~ 72 h later). Similar to what was found in lung cancer cells, JL5 decreased BMP signaling as demonstrated by an increase in *spp-9::GFP* activity (Fig. 5b). A higher concentration of JL5 was needed in comparison to cell cultures experiments to penetrate the *C. elegans* tough outer cuticle covering. Given this conservation of function, we asked whether JL5 would also lead to a trafficking defect in vivo in the whole animal. Treatment with JL5 for 72H. leads to dramatic changes in the localization and trafficking of *daf-4* (BMPR2) – with the receptor being trapped intracellularly within vesicles (Fig. 5c). These studies suggest that JL5 decreases BMPR2 signaling and influences trafficking of the BMPR2 and its mechanism of action likely works through a conserved trafficking pathway.

### BMP inhibitors increase the production of ROS and DNA-DSB)

**Fig. 6:**
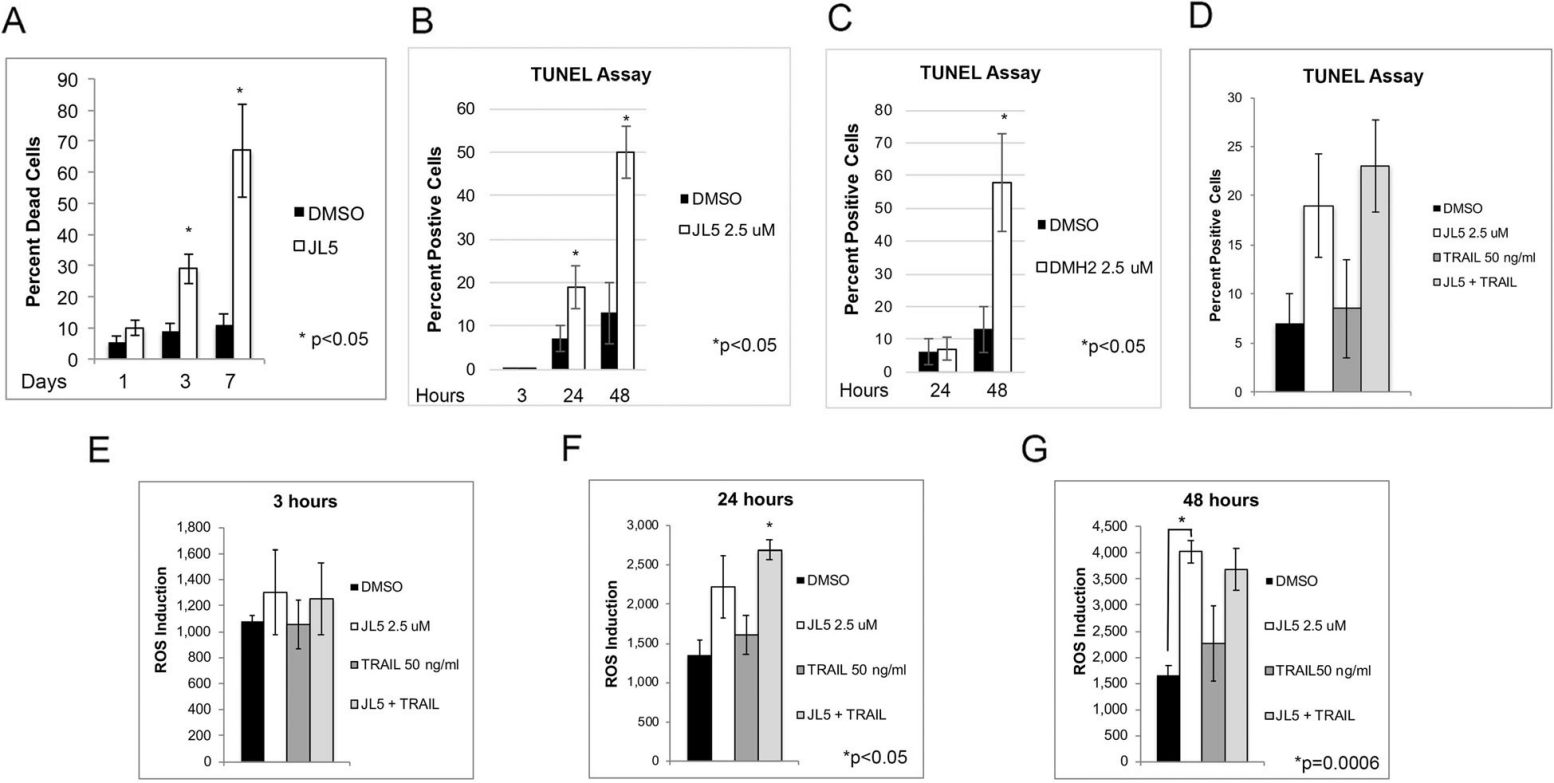
BMP inhibitors increase DNA double-stranded break (DSB) and the production of ROS. **a** H1299 cells treated with 2.5 μM JL5 demonstrate an increase percentage of dead cells over time. **b**-**c** The percentage of H1299 cells with DSB after treatment with JL5 and DMH2 was determined using the TUNEL Assay. JL5 and DMH2 both increased the percentage of cells with DSB over time. **d** After 24 h, TRAIL effects on DSB was not effected by the addition of JL5. **e**-**g** ROS production from H1299 cells treated with JL5 and TRAIL alone and in combination over time. JL5 but not TRAIL increased ROS production. JL5 did not enhance TRAIL induced ROS production. In each set of studies the data represents the mean of at least 4 independent experiments

Dysfunction of the mitochondria and the downregulation of XIAP or TAK1 have both been shown to increase the production of ROS leading to cell death. TRAIL-induced cell death has been reported in some cancer cells to be caused by an increase in ROS production and/or in the formation of DNA-DSB. We found that the induction of cell death induced by JL5 increased over a period of several days (Fig. 6a). We examined whether synergistic cell death with BMP inhibitors and TRAIL involved an increase in ROS and/or DNA-DSB. JL5 increased the percentage of cells with DNA-DSB over a period of 48 h (Fig. 6b). DMH2 has similar inhibition of BMP type I receptors and BMPR2 kinase activity as JL5 and also decreases the expression of XIAP [14, 20]. DMH2 also increased DNA-DSB after 48 h (Fig. 6c). TRAIL did not enhance DNA-DSB induced by JL5 in H1299 cells (Fig. 6d). JL5 increased the production of ROS over a period of 48 h (Fig. 6e-g). TRAIL also did not enhance ROS produced by JL5 (Fig. 6e-g). These studies demonstrate that BMP inhibition does not enhance TRAIL induced cell death by enhancing ROS production and/or DNA-DSB.

### JL5 enhances cell death by the Smac/DIABLO mimetic, AEG40730

**Fig. 7:**
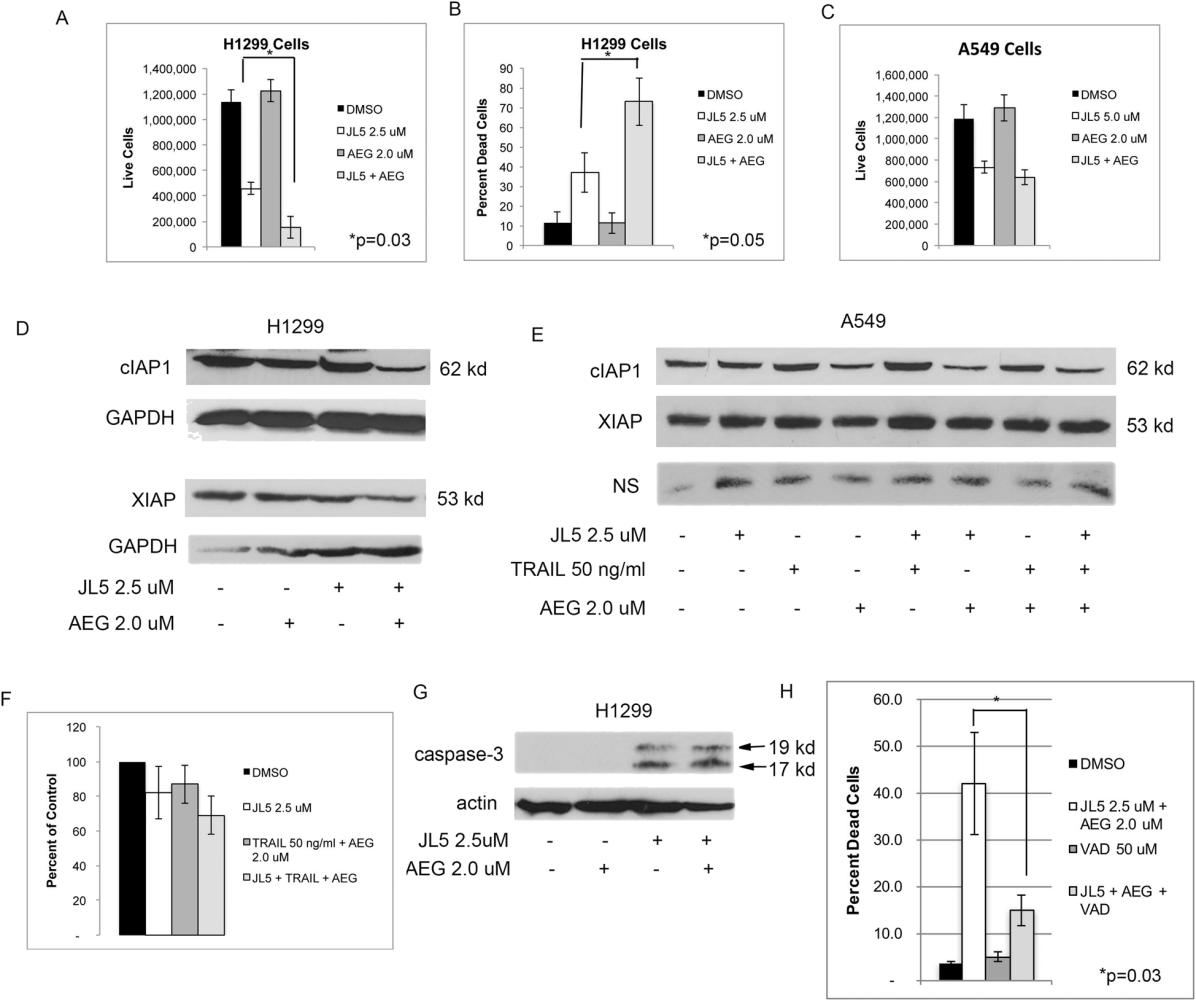
JL5 enhances cell death induced by the Smac/DIABLO mimetic, AEG. **a**-**c** Cells were treated with JL5 and AEG alone and in combination for 48 h and cell counts performed. Studies represent the mean of 4 independent experiments. The Smac mimetic AEG40730 (AEG) by itself had no effect on cell death of the H1299 or A549 cells. JL5 overcame resistance to AEG in the H1299 cells but not the A549 cells. **d** Western blot analysis of H1299 cells treated for 48 h showed that the combination of JL5 and AEG caused a greater decrease in the expression of cIAP1 and XIAP. **e** In the A549 cells, AEG decreased cIAP1 expression but had no effect on the expression of XIAP. NS is a nonspecific band used as a loading control. **f** The A549 cells treated for 48 h were resistant to cell death induced by the combination of AEG, TRAIL, and JL5. **g** Western blot analysis demonstrates cells treated for 48 h with JL5 alone and together with AEG, activated caspase-3 as demonstrated by the production of 17 kDa and 19 kDa fragments. **h** Cell counts of H1299 cells treated for 48 h with JL5 and AEG with and without the pan-caspase inhibitor VAD. VAD significantly decreased cell death induced by JL5 in combination with AED, demonstrating cell death involved the activation of caspases. Studies represent the mean of 4 independent studies

Smac mimetics are small molecules that have been designed to block the binding pocket of Smac/DIABLO on the inhibitor of apoptosis proteins cIAP1, cIAP2 and XIAP. Smac mimetics cause the degradation of cIAP1, cIAP2 and XIAP and induce apoptotic cell death in some cells [43]. Smac mimetics are also reported to prevent the binding of XIAP to the caspases. They have also been shown to overcome TRAIL resistance by blocking XIAP inhibition of caspase-3 [43]. Since the inhibition of XIAP appears to be an important mechanism by which JL5 enhances cell death, we investigated whether JL5 increases cell death induced by Smac mimetics. The Smac mimetic AEG40730 (AEG) [43] by itself had no effect on cell death of H1299 or A549 cells (Fig. 7a-c). JL5 overcame resistance to AEG in H1299 cells but not in A549 cells (Fig. 7a-c). The combination of JL5 and AEG enhanced the downregulation of cIAP1 and XIAP expression in H1299 cells (Fig. 7d). In A549 cells, AEG decreased cIAP1 expression but had no effect on the expression of XIAP (Fig. 7e), or on cell death (Fig. 7f), when used in combination with JL5 and/or TRAIL (Fig. 7f). In H1299 cells, JL5 alone and together with AEG activated caspase-3 (Fig. 7g), while AEG alone did not cause the activation of caspase-3 (Fig. 7g). The pan-caspase inhibitor VAD significantly inhibited cell death induced by JL5 in combination with AEG (Fig. 7h). These studies provide further evidence that inhibition of BMP signaling enhances cell death by cancer therapeutics that induce apoptosis.

## Discussion

The BMP signaling cascade regulates several pro-survival signaling pathways in cancer cells including XIAP, TAK1, and the inhibitor of differentiation proteins (ID1-ID3) [6, 14, 20, 32, 44]. XIAP inhibits caspases by binding activated fragments, inhibiting the processing of executioner caspases into their active forms, and triggering caspase degradation through the ubiquitin proteasome pathway [23, 45]. XIAP inhibition of caspases promotes resistance to many cancer therapeutics including radiation, chemotherapeutics, and TRAIL [28, 35] The inhibition of BMPR2 decreases the expression of XIAP and inhibits the activity of TAK1 [20, 22, 46]. TAK1 is a BMP regulated protein that is a very potent inhibitor of cell death [25, 47, 48]. BMP signaling regulates TAK1 activity, at least, in part by its regulation of XIAP [22]. TAK1 has also been shown to generate resistance to cancer therapeutics [49]. BMP-Smad-1/5 signaling is one of the most potent transcriptional activators of ID1, ID2, and ID3 [17, 18]. ID1-ID3 are also tumorigenic as they stimulate cell survival, proliferation, migration/invasion of cancer cells, inhibition of senescence, and promotion of immortalization of normal cells. Our studies suggest the potential use of BMP small molecule inhibitors to augment cell death of cancer therapeutics that induce cell death by apoptosis.

This is the first report demonstrating that inhibition of a BMP receptor increases cytosolic cytochrome c and Smac/DIABLO. The increased release of cytochrome c and Smac/DIABLO is specific for BMPR2 and not the type 1 BMP receptors. This has significant implications regarding targeting BMP receptors as potential cancer therapeutics. Thus far, all the BMP receptor inhibitors developed target predominantly the type 1 BMP receptors. The BMP receptor inhibitors JL5 and DMH2 have been shown to cause a greater decrease in the expression of the BMP signaling proteins ID1, XIAP, and TAK1, and induce more cell death of lung cancer cell lines than the BMP inhibitors DMH1 and LDN [20]. All these inhibitors have potent inhibition of BMP type 1 receptors but only JL5 and DMH2 inhibit BMPR2 [14]. We hypothesized that the enhanced potency of JL5 in comparison to DMH1 was due to its inhibition of BMPR2. This hypothesis was supported by our studies showing that the knockdown of BMPR2 but not the BMP type 1 receptors decreased XIAP expression and increased cytosolic Smac/DIABLO and cytochrome c. Although JL5 only has an IC50 of 8 μM [14] for BMPR2, it was able to induce the internalization and trapping of BMPR2 in cytoplasmic vesicles, which did not occur with DMH1. BMPR2 must traffick back to the plasma membrane to remain active and trapping in cytoplasmic vesicles leads to inhibtion [39–41]. These studies demonstrate the importance of specifically targeting BMPR2 as a strategy to induce cell death in lung cancer and support further development of more potent BMPR2 inhibitors. Future studies will be needed in animal tumor models to validate this hypothesis.

BMP receptors mediate Smad-dependent and Smad-independent signaling. The cytoplasmic tail of BMPR2 is longer than that of the type 1 receptors, which mediate Smad-independent signaling. XIAP binds the cytosolic tail of BMPR2 and this binding is thought to stabilize XIAP, leading to increased expression [19]. Our studies suggest that BMPR2 regulation of XIAP involves the release of Smac/DIABLO into the cytosol, presumably from the mitochondria where Smac/DIABLO is localized normally. Cytosolic Smac/DIABLO binds and inactivates inhibitor of apoptosis proteins [23]. Cytosolic Smac/DIABLO or Smac3 is also reported to induce the degradation of XIAP and other inhibitor of apoptosis proteins through the ubiquitin proteasome pathway [23, 38]. Prior studies only showed that XIAP was bound to BMPR2 and that the knockdown of BMPR2 resulted in a decreased expression of XIAP. Prior reports did not reveal the mechanisms by which BMPR2 led to the “stabilization” of XIAP. The knockdown of BMPR2 causes an increase in cytosolic Smac/DIABLO, a well-known inhibitor of XIAP. This suggests that BMPR2 regulation of XIAP involves more than just promoting its stabilization. Our studies do not rule out that the downregulation of BMPR2 may also regulate the expression of XIAP through other pathways. The activation of proteolytic cell death pathways cathepsins and/or caspases can also induce the degradation of XIAP [50].

A549 cells have an activating K-ras mutation and are resistant to TRAIL and AEG. They are less responsive to JL5 compared to H1299 cells. Alterations or the inhibition of the TRAIL receptors can prevent the activation of caspase-8 and promote resistance [51]. TRAIL requires the activation of caspase-3 to induce cell death. TRAIL activation of caspase-3 in some cells requires an amplification step at the mitochondria with the release of cytochrome c [51]. TRAIL can also activate caspase-3 in some cells without mitochondrial amplification [51]. XIAP promotes resistance to TRAIL through its inactivation of caspase-3 [51]. In A549 cells, TRAIL did not activate caspase-8 which may be the reason why we did not find enhanced cell death with the combination of TRAIL and JL5. Our data suggests that to enhance TRAIL induced cell death with JL5, there needs to be activation of caspase-8 by TRAIL and a downregulation of XIAP by JL5. In H1299 cells, TRAIL activated caspase-8 and induced increased cytosolic cytochrome c but did not induce cell death. TRAIL did not fully activate caspase-3 as demonstrated by the lack of processing to the 17 kDa fragment. Our studies show that JL5 minimized the resistance to TRAIL by decreasing the expression of XIAP. In A549 cells, JL5 did not decrease XIAP expression, increase cytosolic Smac/DIABLO, or increase cell death. Interestingly, the knockdown of BMPR2 in A549 cells did decrease XIAP expression and increase cytosolic Smac/DIABLO and cytochrome c. It is possible that a more potent small molecule inhibitor of BMPR2 is needed to increase mitochondrial permeability in A549 cells. Genetic alterations of BMPR2 are infrequent in lung adenocarcinomas. Review of the Cancer Genome Atlas of 135 primary lung adenocarcinomas revealed only 3 missense mutations and 1 truncating mutation of BMPR2 [14], suggesting BMPR2 could be targeted with small molecules.

## Conclusions

These studies provide further evidence that the BMP signaling cascade in cancer cells promotes survival. Our studies support that BMP survival mechanisms in cancer cells are mediated predominantly by BMPR2. These data support the development of a more potent and specific BMPR2 inhibitor for potential use to enhance the effects of cancer therapeutics.

## Data Availability

The datasets obtained and analyzed for this study will be made available from the corresponding author in a reasonable request.

## Abbreviations

AEG: AEG 40730
BMP: Bone morphogenetic protein
BMPR: Bone morphogenetic protein receptor
*C. elegans*: *Caenorhabditis elegans*.
c-IAP: inhibitor of apoptosis proteins
DNA-DSB: DNA double stranded breaks
LDN: LDN-193189
NSCLC: Non-small cell lung carcinomas
ROS: Reactive oxygen species
TAK1: Transforming growth factor beta (TGFβ) activated kinase 1
TRAIL: Including tumour-necrosis factor (TNF)-related apoptosis-inducing lingand
VAD: Z-VAD-FMK
XIAP: X-linked inhibitor of apoptosis protein
